# Endotoxin Activity Assay levels correlate with the microbiological results of Gram-negative organisms in septic patients

**DOI:** 10.1186/cc14076

**Published:** 2014-12-03

**Authors:** M Tsunoda, M Kang, M Saito, N Saito, M Namiki, T Harada, M Takeda, A Yaguchi

**Affiliations:** 1 Department of Critical Care and Emergency Medicine, Tokyo Women's Medical University, Tokyo, Japan

## Introduction

The Endotoxin Activity Assay (EAA™; Spectral Diagnostics Inc., Toronto, Canada) is a useful diagnostic test for sepsis due to Gram-negative infection and is based on the reaction of neutrophils to endotoxin complexed with an anti-endotoxin antibody. However, the relations between values of EAA and microbiological data have not been elucidated. Our hypothesis is that EAA values correlate to the results of microbiological cultures and also severity.

## Methods

From July 2008 to July 2013, all adult patients with suspected sepsis admitted to our medico-surgical ICU in whom EAA was measured were included in this study. Data collected included age, gender, ICU mortality, white blood cell (WBC) count, C-reactive protein (CRP), procalcitonin (PCT), EAA levels, SOFA score and results of microbiological culture. Patients with no microbiological data were excluded. Data were analyzed by Kruskal-Wallis test, Mann-Whitney *U *test and multivariate logistic regression. *P *< 0.05 was considered significant.

## Results

Of 569 patients (353 men and 216 women; mean age 66.0 ± 17.4 years), 283 patients had Gram-negative infection and 286 patients had no Gram-negative infection. Of 283 patients with Gram-negative infection, 65 patients had Gram-negative organisms in blood. EAA levels were significantly different between patients with Gram-negative blood, in other infectious sites and no Gram-negative infection (0.45 ± 0.21 vs. 0.39 ± 0.17 vs. 0.36 ± 0.15, *P *= 0.03). The odds ratio (95% confidence interval (CI)) of EAA levels for Gram-negative infection and Gram-negative bacteremia were 3.89 (1.44 to 10.4) (*P *= 0.007) and 3.36 (2.16 to 40.6) (*P *= 0.003), respectively. The odds ratio and CI of age and SOFA score for ICU mortality were 1.03 (1.01 to 1.04) (*P *= 0.0003) and 1.33 (1.26 to 1.41) (*P *< 0.0001), respectively, while gender, WBC, CRP, PCT and EAA levels had no relations with ICU mortality. SOFA score was significantly higher in patients with Gram-negative infection than in patients with no Gram-negative infection (8.0 ± 4.6 vs. 6.7 ± 4.2, *P *= 0.0003).

## Conclusion

EAA levels related to the detections of Gram-negative organisms in cultures. Thus, a high EAA level may show the existence of Gram-negative organisms in patients' sites. EAA levels had relations with SOFA score but no relations with ICU mortality.

